# The association of circulating adiponectin and + 45 T/G polymorphism of adiponectin gene with gestational diabetes mellitus in Iranian population

**DOI:** 10.1186/s40200-015-0156-z

**Published:** 2015-04-16

**Authors:** Mohammad Ali Takhshid, Zinab Haem, Farzaneh Aboualizadeh

**Affiliations:** Diagnostic Laboratory Sciences and Technology Research Center, School of Paramedical Sciences, Shiraz University of Medical Sciences, Shiraz, Iran

**Keywords:** Adiponectin, Gestational diabetes, Iranian population, Single nucleotide, Polymorphism +45 T > G

## Abstract

**Background:**

Adiponectin is an adipokine with insulin-sensitizing effects. We investigated the relationship between the single nucleotide polymorphism (SNP) +45 T > G ( rs 2241766 ;Gly15Gly) in the adiponectin gene, serum adiponectin levels, insulin resistance and risk of gestational diabetes (GDM) in Iranian population.

**Methods:**

65 GDM patients and 70 healthy pregnant women were enrolled in this study. Genotyping for SNP +45 T > G in the adiponectin gene ( rs 2241766 ) was performed by the polymerase chain reaction-restriction fragment length polymorphism method. The level of fasting serum adiponectin, insulin, glucose, and lipid levels were measured. Insulin resistance was estimated using homeostasis model of assessment for insulin resistance (HOMA-IR).

**Results:**

The G allele and TG/GG genotype of rs 2241766 were more frequent than the T allele and TT genotype in GDM patients compared to the controls (p < 0.05). Multiple logistic regression analysis revealed that the risk of GDM was significantly higher in subjects with the TG/GG genotype to those with TT genotype [odds ratio = 2.38, 95% CI 1.09-5.22, p = 0.030]. No significant association was observed between genotypes of rs 2241766 and circulating concentrations of adiponectin. Multiple regression analysis showed that serum adiponectin levels was negatively associated with HOMA-IR in GDM patients (β = −0.385, p <0.01).

**Conclusion:**

The findings demonstrated that TG/GG genotype of rs 2241766 was an independent risk factor of GDM in our population. Furthermore, circulating adiponectin level was negatively correlated with insulin resistance in GDM patients.

## Background

Gestational diabetes mellitus (GDM) is a form of glucose intolerance first diagnosed during pregnancy [1]. The prevalence of GDM in the Iranian population is 4% to 9% of pregnancies [2]. GDM is associated with increased risk of preeclampsia, cesarean section and developing diabetes type 2 (T2D) for the pregnant women, macrosomia and perinatal mortality for the fetus, and brachial plexus injury and hypoglycemia for the neonate [3].

GDM and T2D are multifactorial disorders in which both genetic and non-genetic factors are involved in disease susceptibility and severity. A variety of risk factors including ethnicity, genetics, family history, lifestyle, diet and physical inactivity contribute to the likelihood of developing both T2D and GDM [4]. Obesity is a common risk factor of both T2D and GDM. Obesity could lead to the development of insulin resistance. Many biochemical mediators are suggested to correlate obesity and insulin resistance. Some of these biomarkers are synthesized in the adipose tissue and secreted in the circulation. Leptin, visfatin, resistin and adiponectin are among these mediators [5].

Adiponectin is an adiopkine produced and secreted by adipose tissue. It is involved in regulation of carbohydrate and fatty acid metabolism [6,7]. In addition, it ameliorates inflammation and prevents atherosclerosis [8]. Circulating concentrations of the adiponectin are affected by multiple factors including gender, age and lifestyle [9,10]. It has been shown that circulating adiponectin concentration decreases in T2D, insulin resistance and obesity [11,12]. However, conflicting results have been reported in regards to the circulating adiponectin concentration in GDM [13-18].

ADIPOQ gene which encodes adiponectin is located on chromosome 3q27. This genomic region has revealed strong linkage to insulin resistance and T2D, suggesting that ADIPOQ is a candidate gene for T2D [19]. Several single nucleotide polymorphisms (SNPs) at this locus have been reported, individually or in combination as haplotypes, to be associated with T2D, insulin resistance and obesity in various populations. Studies have focused mostly on SNPs rs17300539 (−11391G > A) and rs266729 (−11377 C > G) in the promoter region, rs 2241766 (Gly15Gly, +45 T > G) in exon 2 and rs1501299 (+276 G > T) in intron 2. These four variants are located within the two adiponectin linkage disequilibrium (LD) blocks: block 1, comprising the promoter sequence spanning the region −14811 to −4120, and block 2, encompassing the exons in the region −450 to +4545. The results of these studies have been controversial with regard to whether variability at this locus has an impact on metabolic phenotypes of T2D and which polymorphisms are responsible for such an effect [20]. SNP +45 T > G is a synonymous mutation (GGT → GGG, Gly15Gly) at exon 2. Yang *et al*. [21] showed that SNP +45 T > G may affect expression of adiponectin by affecting RNA splicing and stability. The literature reveals that the G allele of SNP +45 T > G in the adiponectin gene has an association with obesity, insulin resistance and T2D in several populations even though the findings have been controversial [20].

Recently, very few studies have investigated the associations of SNP +45 T > G in the adiponectin gene with GDM. The results of these studies were controversial with regard to the impact of this polymorphism on the risk of GDM and on its associations with metabolic characteristic of GDM [22-24]. One of the reasons of such controversy might be ethnicity as the studies conducted on the Malaysian and Qatari subjects showed the association of G allele of SNP +45 T > G in the adiponectin gene with the risk of GDM, while in another study carried out on Greek population the results did not reveal such an association. In addition, the previous studies demonstrated different results with respect to the impact of SNP + 45 T > G in the adiponectin gene on circulating level of adiponectin, obesity and insulin resistance. Therefore, we aimed to evaluate the possible association between the SNP +45 T > G in the adiponectin gene and circulating total adiponectin levels with the risk of GDM, obesity and insulin resistance in Iranian pregnant women.

## Methods

A sample of 135 unrelated Iranian pregnant women was enrolled as the study group. The pregnant women were classified as non-diabetic control group (N = 70) or having GDM (N = 65) according to the results of oral glucose tolerance test (OGTT). The GDM diagnosis was performed based on American Diabetes Association 2009 criteria, between the 24-28th week of gestation [25]. Maternal BMI (kg/m^2^) was calculated as the ratio of the weight (kg) to the squared height (m). The absence of family history for the T2D, absence of clinical evidence of any major disease and absence of medication use that may alter glucose tolerance were inclusion criteria for the control pregnant women. The inclusion criteria for the pregnant women with GDM were [1]: newly diagnosed cases [2], no previous use of oral hypoglycemic agents. The exclusion criteria of the study were the presence of type-1 or type-2 diabetes mellitus and other known major diseases. The study protocol was approved by the ethics committee at Shiraz University of Medical Sciences, Shiraz, Iran. Informed written consent was obtained from all the participants.

### Biochemical determinations

Fasting venous maternal blood sample was collected in both groups, after an overnight 12- hour-fasting between 8:30 and 9:30 am. The sera were separated immediately and stored at −70°C until biochemical analyses were performed. Fasting blood glucose (FBG), total cholesterol (TC), triglyceride (TG) and high-density lipoprotein cholesterol (HDL-C) were measured by using commercially available kits. Fasting plasma low-density lipoprotein cholesterol (LDL C) was calculated using the formula of Friedewald. HbA1C was measured by ion-exchange high performance liquid chromatography. Serum insulin concentration was measured by radioimmunoassay applying available commercial kits. Serum total adiponectin level was measured by the immunoassay method using a commercially human adiponectin ELISA kit (Biovendor, Czech Republic) according to the manufacturer’s instructions. The lowest detectable level of serum adiponectin was 0.5 μg/ml and intra- and inter-assay coefficients of variation were 4.2% and 9.5%, respectively.

### Insulin resistance and sensitivity indices

In this study, we used the QUICKI (quantitative insulin sensitivity check index) model and HOMA-IR (homeostasis model of assessment for insulin resistance) for the evaluation of insulin sensitivity. These models were correlated with direct measurement of insulin sensitivity using the euglycemic-hyperinsulinemic clamp and validated for evaluation of insulin sensitivity in GDM. The QUICKI was calculated by the following formula: 1/ (log [fasting insulin] + log [fasting glucose]). HOMA-IR is defined as follows: ([fasting glucose] × [fasting insulin])/22.5 [26].

### SNP 45 T/G genotyping

Genomic DNA was extracted and stored at −70 C prior to polymerase chain reaction (PCR). Genotyping for detection of SNP 45 T/G in the adiponectin gene was performed using PCR-RFLP (restriction fragment length polymorphisms) method. In brief, a DNA fragment corresponding to the Adipo45T/G was amplified by forward primer (5′–GAAGTAGACTCTGCTGAGATGG–3′) and reverse primer (5′–TATCAGTGTAGGAGGTCTGTGATG–3′). For PCR reactions 0.4 μM of each primer were combined with 50 ng of DNA, 0.25 mM dNTPs,1.5 mM of MgCl2, and 1.0 U Taq DNA polymerase (Cinagene, Iran) in total volume of 25 μl. PCR thermal cycling was started with initial denaturation at 95°C for 5 min, followed with 35 cycles of amplification which included denaturation at 95°C for 30 sec, annealing at 58°C for 30 sec, extension at 72°C for 30 sec, and final extension at 72°C for 10 min. PCR products were digested by SmaI restriction enzyme (Fermentas Life Sciences, Lithuania) and separated on 2% agarose gel electrophoresis. The TT homozygous genotype was marked by a single 372 bp (undigested) fragment, TG heterozygous genotype was digested into 3 fragments of 372 bp, 209 bp and 163 bp, and GG homozygous genotype was digested into 2 bands of 209 bp and 163 bp . In order to evaluate the reproducibility of the genotyping procedure, duplicate genotyping reactions were performed on 10% of the samples.Genotyping success rate was >99%.

### Statistical methods

All statistical analyses were performed using SPSS 15.0 software (SPSS Inc., Chicago, IL, USA). The normal distribution of variables was assessed by the Shapiro–Wilk test. Student’s t-test and Mann–Whitney U-test were used to compare the normally distributed and not normally distributed variables, respectively. The data were presented in mean ± standard deviation (SD) or ratio and percent. Multiple stepwise linear regression analysis was conducted to assess the association of serum adiponectin and biochemical and demographic variables. The differences across various continuous parameters of adiponectin tertiles were computed using analysis of variance (ANOVA). χ^2^ test was used to compare the frequencies of allele and genotypes. Logistic regression analysis was used to examine the independent association of the SNP +45 T > G in the adiponectin gene with GDM. Confounding variables including maternal BMI, maternal age, and maternal HOMA-index were included in the model. Multivariate analysis controlling for the potential confounders including age, and HOMA-ir was used to compare the means of circulating adiponectin and BMI between different genotypes of rs 2241766. For all comparisons, p < 0.05 was considered as statistically significant.

## Results

### Comparison of baseline characteristics between subjects with and without GDM

The baseline demographic and serum biochemical measurements of patients with GDM and age-matched healthy pregnant women are shown in Table 1. As seen in Table 1, there were no significant differences between demographic characteristics of GDM and non-GDM subjects. Insulin resistance index (as assessed by HOMA-IR) (P < 0.05), FPG (P < 0.01) and HbA1C (P < 0.001) were higher in GDM patients compared to non-GDM subjects. Circulating adiponectin concentration was similar in the GDM group (12.5 ± 5.6, ranged from 4.6 to 28.6 μg/ml) and non-GDM subjects (13.3 ± 7.4, ranged from 2.5 to 27.0 μg/ml). Blood lipids concentrations were also similar in GDM and non-GDM subjects. Serum insulin levels tended to be higher in the GDM subjects compared with the non-GDM ones, but it was not statistically significant.

**Table 1:** **Demographic characteristics and biochemical measurements in the GDM patients and Non- GDM subjects**

	**Non GDM group (n = 70)**	**GDM group (n = 65)**	**p**
**Maternal age(y)**	28.4 ± 3.7	29.2 ± 3.1	0.24
**Gestational age (week)**	29.3 ± 3.2	29.8 ± 3.4	0.55
**BMI(Kg/m** ^**2**^ **)**	27.6 ± 3.7	29.0 ± 5.0	0.12
**FPG(mg/dl)**	78 ± 12	91 ± 27	0.002
**HbA1C (%)**	5.1 ± 0.4	5.8 ± 1.0	<0.001
**Insulin( μIU/ml)**	16.3 ± 6.7	18.4 ± 13.1	0.33
**HOMA- IR**	3.2 ± 1.4	4.2 ± 3.1	0.036
**QUICKI**	0.33 ± 0.03	0.32 ± 0.03	0.15
**TG(mg/dl)**	264 ± 91	258 ± 82	0.73
**TC(mg/dl )**	240 ± 53	221 ± 50	0.08
**HDL-C(mg/dl )**	52 ± 12	50 ± 11	0.28
**TG/HDL**	5.4 ± 2.4	5.6 ± 2.6	0.66
**LDL-C(mg/dl )**	120 ± 27	111 ± 29	0.13
**Adiponectin (μg/ml )**	13.3 ± 7.4	12.5 ± 5.6	0.59

BMI: body mass index. FPG: fasting plasma glucose. HbA1c: hemoglobin A1c. HDL-C: high-density lipoprotein cholesterol. LDL-C: low-density lipoprotein cholesterol. HOMA-IR: homeostasis model assessment index for insulin resistance. QUICKI: quantitative insulin sensitivity check index. Values are presented as mean ± SD. Student- t test was used for comparing data between groups.

### Correlation between serum adiponectin and clinical parameters

Pearson and Spearman correlation analysis showed that circulating total adiponectin concentration had a negative correlation with age (r = −0.293; *p* < 0.03), BMI (*r* = −0.288; *p* = 0.032), HOMA (*r* = −0.353; *p* = 0.011) and TG (*r* = −0.277; *p* = 0.038). Conversely, adiponectin showed a positive relationship with HDL cholesterol (*r* = 0.311; *p* = 0.022). A stepwise multiple regression model was then applied and the results revealed that HOMA-ir and age (P < 0.009 and P < 0.022, respectively) were independent predictors of circulating adiponectin. HOMA-ir accounted for 12.4% of the variance, whereas 10.9% was obtained for age. The other analyzed variables did not show any contribution to the regression model (Table 2).

**Table 2:** **Multiple regression analysis with stepwise analysis for serum adiponectin levels as dependent variable**

**Parameters**	**Model 1**	**Model 2**
**BMI (Kg/m** ^**2**^ **)**	-0.132(0.468)	-0.163(0.345)
**TG (mg/dl )**	-0.245(0.101)	-0.144(0.348)
**HDLC (mg/dl )**	0.227(0.145)	0.083(0.622)
**Age (year)**	--------	-0.330(0.024)*
**HOMA-ir**	-0.353(0.022)*	-0.385(0.009)*
**R** ^**2**^	0.124	0.233

Data are β (*P* value). Parameters from model 1 and model 2 includes BMI(Kg/m^2^), triglyceride(TG), HDL cholesterol(HDLC), HOMA-ir, In model 2, age of patients was added to the parameters of the model 1. *R* ^2^, multiple coefficient of determination; β, standard correlation. In each model, all patients were included.

The relationship between adiponectin tertiles and various biochemical characteristics has been shown in Table 3. GDM patients with lowest serum adiponectin tertile (<9.3 μg/ml) had a significantly higher serum insulin concentration and HOMA-IR in comparison with those who were in the second tertile (9.3-13.9 μg/ml) and third tertile (<13.9 μg/ml) (*P* < 0.05). They also had a lower HDL- cholesterol level and QUICKI (*P* < 0.05) (Tables 3). No significant difference was found between second tertile and third tertile groups.

**Table 3:** **Relationship between serum adiponectin tertiles and various biochemical characteristics in the GDM patients**

	**Tertile 1(< 9.3)**		**Tertile 2 (9.3-13.9)**		**Tertile3 (>13.9)**		**P values T1 V T3**
	**Mean ± SD**	**95%CI**	**Mean ± SD**	**95%CI**	**Mean ± SD**	**95%CI**	
**Age(y)**	29.9 ± 2.6	28.3- 31. 6	29.6 ± 3.1	27.4- 30.1	28.2 ± 2.7	27.1- 30.3	0.134
**Gestational age(week)**	30.1 ± 3.9	27.6- 32.5	29.7 ± 2.7	28.5- 31.5	29.5 ± 3.9	27.0- 31.3	0.673
**BMI(Kg/m** ^**2**^ **)**	31.4 ± 3.9	27.6- 35.2	28.4 ± 4.8	25.9- 31.7	27.8 ± 4.3	25.4- 29.1	0.073
**FPG(mg/dl)**	104 ± 41	78 -130	86 ± 15	76- 93	86 ± 16	79- 97	0.082
**HbA1C (%)**	5.9± 1.4	4.8-7.0	5.7 ± 0.7	5.4- 5.9	5.9 ± 0.9	5.5- 6.5	0.794
**Insulin( μIU/ml)**	26.7 ± 21.0	13.4-39.9	14.2 ± 4.9	11.6- 16.8	16.2 ± 7.6	11.8- 20.6	0.037
**HOMAIR**	6.4 ± 4.7	3.4- 9.4	3.0 ± 1.2	2.4- 3.7	3.5 ± 1.8	2.5- 4.6	0.01
**QUICKI**	0.30 ± 0.02	0.29-0.32	0.33 ± 0.02	0.32- 0.34	0.33 ± 0.04	0.31- 0.35	0.016
**TG(mg/dl)**	267 ± 88	212-323	255 ± 65	219- 286	251 ± 98	197- 312	0.624
**TC(mg/dl )**	196 ± 46	167-225	232 ± 52	208- 261	231 ± 48	199- 257	0.066
**HDL-C(mg/dl )**	47 ± 10	40-53	48 ± 11	43- 57	55 ± 12	46-58	0.049
**LDL-C(mg/dl )**	104 ± 29	86-123	116 ± 29	101- 132	112 ± 30	94- 129	0.478
**TG/HDL**	6.4 ± 3.3	4.3-8.5	5.5 ± 2.5	4.2- 6.8	5.2± 2.2	3.9- 6.4	0.026
**Adiponectin (μg/ml)**	6.9 ± 1.6	5.9-8.0	11.2 ± 1.4	10.5- 11.8	18.8 ± 4.5	16.3- 21.4	<0.001

Values are presented as mean ± SD and 95%CI (***95*** **%** ***confidence interval***). The differences across various parameters of adiponectin tertiles were tested using ANOVA and least significant ***difference*** **(** ***LSD*** **)** as ***post hoc tests***.BMI: body mass index. HbA1c: hemoglobin A1c. HDL-C: high-density lipoprotein cholesterol. LDL-C: low-density lipoprotein cholesterol. HOMA-IR: homeostasis model assessment index for insulin resistance. QUICKI: quantitative insulin sensitivity check index.

### Genotype and allele analysis

The genotype and allele frequencies in the non-GDM subjects are shown in Table 4. Genotype distributions of SNP +45 T > G in the adiponectin gene for both GDM and non- GDM subjects did not deviate from Hardy-Weinberg equilibrium (p <0.05). The findings showed that there was a significant association between genotypes and alleles of SNP 45 T/G and GDM. The GT genotype (p = 0.012, odds ratio = 2.55, 95% CI; 1.21-5.37) and G allele (p = 0.025, odds ratio = 2.13, 95% CI 1.09- 4.15) were more common in the GDM patients compared to the non-GDM group (Table 4).

**Table 4:** **Genotype and allele frequencies of adiponectin gene SNP T45G in the non –GDM (control) and GDM patients**

		**Genotype frequency**			**Allele frequency**		
**group**	**n**	**TT**	**GT/GG**	**p**	**T**	**G**	**p**
**Non GDM**	70	54(77.2%)	16(22.8%)	0.012^*^	124(88.6%)	16(11.4%)	0.025^*^
**GDM**	65	37(56.9%)	28(43.1%)		102(78.5%)	28(21.5%)	
		OR (95% CI), 2.55 (1.21-5.37)			OR (95% CI), 2.13 (1.09 -4.15)		

Genotypes are reported as number with percent in parentheses. *Two**-**tailed chi-square test (χ^2^). 95%CI = 95% confidence interval.

Logistic regression analysis was conducted to examine whether the SNP +45 T > G was independently associated with GDM. The findings showed that subjects with the G-allele of SNP +45 T > G compared to subjects with TT genotype had 2.55-fold higher risk for GDM (odds ratio = 2.55, 95% CI; 1.21-5.37, p = 0.013). The results remained significant when confounding factors including BMI, age and HOMA-index of mothers entered the model (odds ratio = 2.38, 95% CI; 1.09-5.22, p = 0.030).

### Effect of SNP +45 T > G in the adiponectin gene on circulating adiponectin concentrations and BMI

In multivariate analysis which was controlled for the potential confounders including age, and HOMA-ir, no significant differences were observed in genotypes of SNP +45 T > G with respect to BMI (TT = 28.6 ± 4.9, GT/GG = 29.6 ± 5.5 Kg/m^2^, p = 0.77) and circulating concentrations of total adiponectin (TT = 13.1 ± 4.7, GT/GG = 11.7 ± 6.4 μg/ml, p = 0.57).

## Discussion

This study was performed to evaluate the possible association between the +45 T > G (rs2241766) in the ADIPOQ gene, circulating adiponectin level, insulin resistance, and metabolic characteristics of GDM in Iranian population. The findings demonstrated that the GT/GG genotype and G-allele of SNP +45 T > G in the adiponectin gene were more frequent in the GDM patients than in the non-GDM subjects. The results also revealed that HOMA-ir and age are independent predictors of serum adiponectin levels in the GDM patients. To the best of the researchers’ knowledge, this is the first study that has been conducted on the Iranian population in which the association of SNP +45 T > G in the ADIPOQ gene with GDM was addressed.

The results of previous studies show ethnic variations in allelic and genotypic frequencies of SNP +45 T > G in the adiponectin gene [20]. In this study, the frequency of TG/GG genotype and G allele among the non-GDM subjects was approximately 23% and 11%, respectively which is in line with Namvaran, et al.’s findings [27]. The frequency of the rare G allele observed in the control group of this study is similar to that reported for the healthy subjects in some other populations [see for review 31] but it is lower than that reported for the healthy Chinese and Korean subjects (30% and 20%, respectively) [28,29].

The association of SNP +45 T > G in the adiponectin gene with T2D and insulin resistance has extensively been investigated in several populations including the Iranian ones There is no consistency among these studies; however, more studies have indicated that SNP +45 T > G in the adiponectin gene is associated with insulin resistance and T2D [20].The association of adiponectin gene polymorphisms and GDM has been evaluated in recent studies [22-24] and controversial results have been obtained. With regard to the association of SNP +45 T > G in the adiponectin gene with risk of GDM, the findings of logistic regression analysis showed that the risk of GDM was 2.5 fold higher in the subjects with GT/GG genotype of SNP +45 T > G in the adiponectin gene compared to those with TT genotype. This association remained, even after adjusting for confounding factors including maternal age, insulin resistance index and HbA1c. These results are consistent with Low et al.’s study which was conducted on a Malaysian population [22]. The current study did not show any association between SNP +45 T > G in the adiponectin gene and circulating adiponectin which is inconsistent with the findings of Low et al.’s study [22]. They found that the subjects with TG/GG genotype had a lower circulating adiponectin concentration. In another study, Rizk et al. [24] investigated the association of two SNPs of adiponectin gene (+45 T/G and +276 G/T) and GDM. The results indicated an association between GG genotype of SNP +45 T > G and GDM among Arab residents in Qatar. They also found that there was no difference in the circulating adiponectin levels between GDM patients with different genotypes of SNP +45 T > G. Recently, Beltcheva et al. [23] examined the association of three common SNPs of adiponectin gene (−11377 C/G, +45 T/G and +276 G/T) and GDM. The results revealed an association between SNP +276 G/T and GDM, but not with others. Differences in demographic characteristics, genetic background, environmental factors and diagnostic criteria for diagnosis of GDM could account for the inconsistency among the studies.

Given the fact that SNP +45 T > G is a synonymous mutation (Gly15Gly), the exact molecular mechanisms which would be responsible for association of SNP +45 T > G with GDM and T2D are still unknown. A possible mechanism is the effects of this silent mutation on expression of ADIPOQ gene and circulating concentration of adiponectin. It has been reported that SNP +45 T > G in the adiponectin gene could affect the expression of adiponectin by influencing adiponectin mRNA processing or stability [21]. It has also been reported that SNP +45 T > G in the adiponectin gene has an association with circulating levels of adiponectin [28,30]. However, our results in accordance with data from other studies [31] showed that there are no association between circulating level of adiponectin, and SNP +45 T > G genotypes. Linkage disequilibrium of SNP +45 T > G with other functional SNPs in the adiponectin gene, particularly SNP +276 G > T, variation in ethnic background, environmental factors, sample size and the type of study may account for the discrepancies across studies.

Some previous studies have also found an association between SNP +45 T > G in the adiponectin gene and obesity both in healthy individuals and T2D patients [32,33]. In this study, BMI was used to represent the degree of obesity. Nevertheless, the data revealed no significant association of SNPs +45 and BMI in GDM and non-GDM subjects. The results were in line with Krizova et al. and Hasani-Ranjbar et al.’s studies [34,35]. It could be inferred from the literature that differences of race, the sum of the sample, complicated diseases, etc. would be responsible for these differences.

Data regarding circulating adiponectin concentration and its association with metabolic characteristics of GDM are also controversial. Some previous studies have found decreased circulating adiponectin concentrations in the GDM patients than non-GDM control subjects. Renheim et al. [13] showed lower circulating adiponectin concentration in the GDM group. Additionally, they found a reduction in adiponectin mRNA levels in adipose tissue biopsies of GDM subjects. Williams et al. [14] found that serum adiponectin concentrations were lower in women with GDM than controls. Furthermore, they showed that women with low level of serum total adiponectin (below 6.4 mg/ml) at first trimester of gestation had increased risk of GDM as compared to pregnant women with higher concentrations of the adiponectin. Lain et al. [36] showed that women with hypoadiponectinemia(less than the 25th quartile) were 11 times more likely to develop GDM, and it continued after controlling BMI. Kinalski et al. [15] did suggest that hypoadiponectinaemia in GDM may reflect impaired glucose metabolism during pregnancy. Thus, on the basis of the findings, it has been speculated that reduced adiponectin levels at the beginning of pregnancy could be considered a risk factor for development of GDM. In a recent publication on 40 GDM and 40 non-GDM subjects, they showed that the level of adiponectin in the GDM patients was significantly lower in healthy pregnant women at early pregnancy (10–12 weeks) than those in late pregnancy (36–38 weeks), and the level of adiponectin was negatively correlated with HOMA-IR. In contrast to the above-mentioned down-regulated adiponectin levels in GDM, other studies show unchanged circulating adiponectin levels in patients with GDM compared to the non-GDM women. Recently, Saucedo et al. [17], in a case control study performed on 60 GDM and 60 normal glucose tolerance (NGT) controls, demonstrated that circulating adiponectin levels are similar in both groups although the GDM patients had a greater insulin resistance than normal pregnant womwn. Paradisi etal [18] also reported similar findings. They examined serum concentrations of adiponectin and insulin resistance in 50 high-risk women at first, second and third trimesters of pregnancy. They showed a progressive decline of plasma adiponectin levels as the pregnancy advanced in both normal glucose tolerance (NGT) controls and GDM subjects; however, values observed in NGT and GDM subjects were similar. In the current study, circulating total adiponectin concentration was lower among GDM subjects although the difference was not statistically significant. Serum total adiponectin concentration in the GDM patients negatively correlated with age, HOMA-IR, TG and positively associated with serum HDL cholesterol concentration. The stepwise multivariate regression analysis revealed that age and HOMA-ir significantly and independently contributed to the serum adiponectin values in patients with GDM. These variables jointly accounted for 23.3% of the variance. When the patients were classified into 3 tertile of adiponectin, we observed that those patients with lower adiponectin had a higher IR (higher HOMA and lower QUICKI). These inconsistencies could be related to different criteria for diagnosis of GDM, gestational age, differences in ethnic backgrounds of the subjects, and high inter-subject variability in basal adiponectin concentration [36,37]. Variation in dietary intakes specifically magnesium and plant-based foods [10], the methods used to measure adiponectin, and sample size may also be related to this discrepancy.

Our study had some limitations. The major limitation was the relatively small sample size for testing the associations of particular SNP with the disease phenotype. In spite of this limitation, we could detect the association between SNP +45 G > T of ADIPOQ gene and GDM. Another limitation was that we tested only one SNP of ADIPOQ gene that limited the possibilities to examine a wide range of haplotype combinations. Therefore, further investigations with a larger sample size are required to clarify the impact of SNP +45 T > G in the adiponectin gene on the risk of GDM and different forms of circulating adiponectin with GDM. Finally, in this study, we considered only the total concentrations of serum adiponectin while in human plasma, adiponectin circulates in trimeric, hexameric and oligomeric forms which might be responsible for adiponectin insulin sensitizing effects [38].

## Conclusion

The findings of this study indicated that serum adiponectin was negatively correlated with insulin resistance in GDM patients. Furthermore, SNP +45 T > G in the adiponectin gene was associated with the susceptibility to GDM in our population. Further studies are needed to explore the potential mechanisms by which SNP +45 G > T modulates susceptibility to GDM.
